# Salycilic Acid Induces Exudation of Crocin and Phenolics in Saffron Suspension-Cultured Cells

**DOI:** 10.3390/plants9080949

**Published:** 2020-07-28

**Authors:** Azar Moradi, Fatemeh Zarinkamar, Stefania De Domenico, Giovanni Mita, Gian Pietro Di Sansebastiano, Sofia Caretto

**Affiliations:** 1 Department of Plant Science, Faculty of Biological Science, Tarbiat Modares University, Tehran 14115, Iran; azar.moradi@modares.ac.ir (A.M.); zarinkamar@modares.ac.ir (F.Z.); 2 Institute of Sciences of Food Production (ISPA-CNR), 73100 Lecce, Italy; stefania.dedomenico@ispa.cnr.it (S.D.D.); giovanni.mita@ispa.cnr.it (G.M.); 3 DiSTeBA (Department of Biological and Environmental Sciences and Technologies), University of Salento, 73100 Lecce, Italy

**Keywords:** *Crocus sativus* L., cell suspension, crocin, salicylic acid, subcellular compartmentalization, antioxidant activity

## Abstract

The production of crocin, an uncommon and valuable apocarotenoid with strong biological activity, was obtained in a cell suspension culture of saffron (*Crocus sativus* L.) established from style-derived calli to obtain an in-vitro system for metabolite production. Salycilic acid (SA) was used at different concentrations to elicit metabolite production, and its effect was analyzed after a 4 days of treatment. HPLC-DAD analysis was used for total crocin quantification while the Folin-Ciocâlteu method was applied for phenolic compounds (PC) content. Interestingly, despite cell growth inhibition, a considerable exudation was observed when the highest SA concentration was applied, leading to a 7-fold enhanced production of crocin and a 4-fold increase of phenolics compared to mock cells. The maximum antioxidant activity of cell extracts was evidenced after SA 0.1 mM elicitation. Water-soluble extracts of saffron cells at concentrations of 1, 0.5, and 0.1 µg mL^−1^ showed significant inhibitory effects on MDA-MB-231 cancer cell viability. The heterologous vacuolar markers RFP-SYP51, GFPgl133Chi, and AleuRFP, were transiently expressed in protoplasts derived from the saffron cell suspensions, revealing that SA application caused a rapid stress effect, leading to cell death. Cell suspension elicitation with SA on the 7th day of the cell growth cycle and 24 h harvest time was optimized to exploit these cells for the highest increase of metabolite production in saffron cells.

## 1. Introduction

Saffron, *Crocus sativus* L., representing the most expensive spice in the world [1], is mostly used in medicine because of its wide pharmaceutical properties such as anti-depressive, antitussive, antigenototoxic, anticonvulsant, antihypertensive, anti-Alzheimer’s, antioxidant, anti-diabetic, anti-Parkinson’s, antinociceptive, and anti-inflammatory activities [2]. Saffron is valorized by crocin (crocetin ester), picrocrocin, and safranal bioactive compounds, the three main bioactive compounds of saffron′s stigmas [3]. Crocin, the first most abundant compound of saffron, is responsible for the red color and is the highest and only water-soluble carotenoid, serving as a natural food colorant and an antioxidant even stronger than α-tocopherol [4,5]. Recently, crocin effects have proven to be responsible for most of the saffron medicinal effects [6]. Among other effects, it had anti-depressive effects on subjects with metabolic syndrome [7]; its application on rat hippocampus also improved memory function and learning [8]. Unfortunately, the supply of crocin remains expensive. Plant secondary metabolite production by cell cultures is gaining attention as a reliable, safe, and continuous supply strategy alternative to chemical synthesis [9]. The natural variability occurring in plant in-vitro cultures, although often unwanted, could be exploited for identifying interesting biosynthetic variants [10]. Moreover, the production of bioactive metabolites through cell cultures may be advantageous when the metabolites are biosynthesized in specific plant tissues [11]. Elicitor application to plant cell cultures has been proved to successfully enhance the biosynthesis of the desired bioactive compounds. In particular, it was shown with phytosterol and tocopherol in mung beans, safflower, and sunflower [12,13], artemisinin in *Artemisia annua* [14] or taxol in *Taxus baccata* suspension cultures [15]. Establishing such a bioproduction strategy for crocin, using a cell suspension culture and applying an elicited induction of biosynthesis, can be considered as a relevant improvement in satisfying the great demand for this valuable pigment in the food industry.

Salicylic acid (SA), known to be a plant defense signaling compound, has a pivotal role in alleviating injuries due to abiotic and biotic stress in plants [16,17]. SA was also shown to act as an elicitor to induce secondary metabolites accumulation. The effects of SA on the biosynthesis of several metabolites have been extensively clarified in many plants [18]. Phenolic compounds (PC) were elicited in cell suspension cultures of *Thevetia peruviana* [19] and *Polygonum multiflorum* [20]; similarly, alkanes and fatty acids were elicited in cell cultures of *Jatropha curcas* [21].

In our recent work, callus cultures were established from two different corm and style explants of saffron [22]. Applying different plant growth regulators (PGR), we optimized crocin production in style-derived calli, using thidiazuron in combination with naphthalene acetic acid on MS medium. In this study, style-derived calli with the best crocin production were used to establish a saffron cell suspension culture. The influence of different concentrations of SA was studied on cell growth, and on the accumulation of crocin and PC content in saffron cell extracts or their corresponding spent media. The extracts were further tested for their antioxidant activity and potential effect on cancer cells. To better interpret the effects of SA on cells, their compartmentalization was investigated microscopically in vivo using saffron protoplasts transiently transformed with different vacuolar markers such as RFP-SYP51, GFPgl133Chi, and AleuRFP. Altogether, our results provide a wide characterization to support a simple but effective optimization of the crocin bioproduction process.

## 2. Results

### 2.1. Cell Suspension Culture and SA Effects on Cell Growth

**Figure 1:**
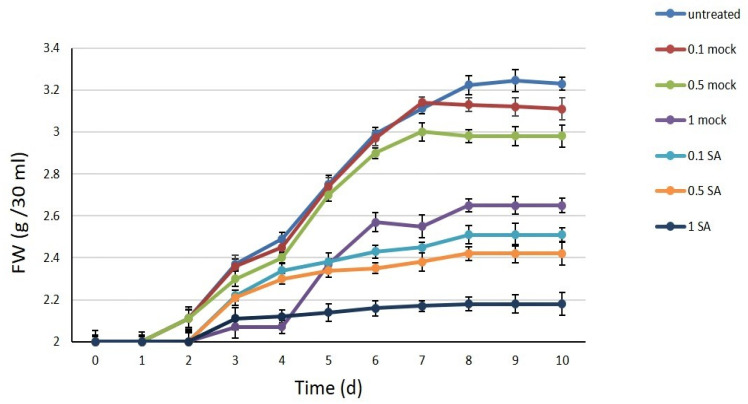
Growth curve of saffron cell cultures within 10 days. Untreated, without methanol or salicylic acid (SA); mock 0.1, 0.5, and 1; SA 0.1, 0.5, and 1 mM treated cells. Values are reported as the average of three replicates ± standard deviation.

The saffron cell suspension was obtained from optimized friable calli [22] and a 10-day subculture cycle was optimized in a few months. Methanol-dissolved SA at different concentrations (0.1, 0.5, and 1 mM) was administered to saffron cells. The same amount of methanol used for the preparation of each SA concentration was applied to saffron control cells and referred to as 0.1, 0.5, and 1 mock, while cells without methanol or SA represented controls. The effects of these treatments on cell growth were evaluated by measuring the fresh weight and shown in Figure 1. Three days after subculturing, the fresh weight of untreated control cells began to increase until day seven when the stationary phase started. SA negatively affected the growth rate of treated cells compared to the mock cells; the inhibitory effect of SA 1 mM on the cell growth rate was remarkable, showing just 0.2 g increased biomass after 10 days of subcultivation. The doses of 0.1 and 0.5 mM SA also showed an inhibitory effect on growth, but no significant difference was evidenced between them. Further, mock 1 showed a significant decrease in growth rate with a shorter log phase and a slight increase in fresh weight, while mock 0.5 and 0.1 showed a pattern similar to the untreated cells.

### 2.2. Analysis of Crocetin Esters and PC Profile Following the Imposition of SA

**Figure 2:**
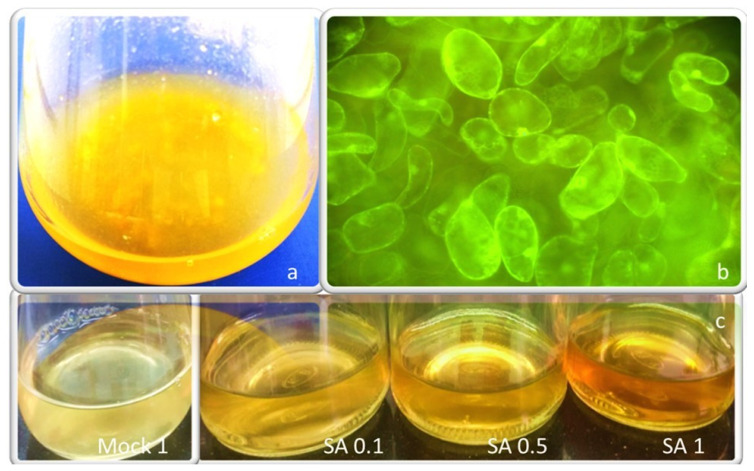
Established saffron cell suspension cultures from style explant in MS medium containing 3 mg L^−1^ NAA and 1 mg L^−1^ TDZ (**a**); cell viability of untreated (control) saffron suspension culture after staining with fluorescein diacetate (**b**); increasing coloration of the spent medium of saffron treated-cells with increasing SA concentrations (**c**).

**Figure 3:**
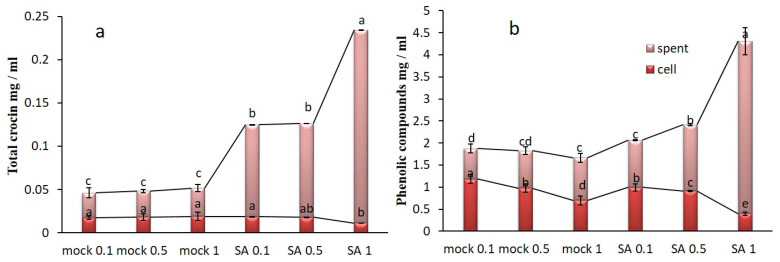
Total crocin (**a**) and PC (**b**) content in control and SA treated cells in saffron cell suspension cultures. Every column represents the total crocin and PC in cells and spent media. Common letter in each group indicates no significant differences (*p* < 0.05).

An increase of the characteristic pigmentation of in-vitro saffron cell cultures in the spent medium was observed soon after treatment with SA 0.1, 0.5, and 1 mM (Figure 2). It is generally considered that a few days are required for a convenient metabolite accumulation but the color intensity is not a reliable quantitative measurement when dealing with metabolites with different oxidation forms and optical characteristics. For this reason, we measured molecular species more precisely. The total crocetin esters (glycosylated pigments) were evaluated in saffron cell cultures as previously published by our team [22]. In the present study, the production of total crocin (from 2 g FW incubated cells were quantified as mg mL^−1^) was measured, quantifying concentration in cell extracts as well as in the spent medium. Along with increasing SA concentrations, total intracellular crocin content decreased, but crocin content in the spent medium increased, reaching a maximum value of 0.22 mg mL^−1^ at SA 1 mM, approximately 5-fold higher than the mock 1 (0.032 mg mL^−1^). Moreover, 0.1 and 0.5 mM SA induced a significant increase of crocin production compared to the mock 0.1 and 0.5, while no significant difference was found between them (Figure 3a). Similarly, by increasing SA concentration, the intracellular PC decreased significantly compared to the mock cells, showing the highest contents at SA 0.1 mM mock cells (1.18 mg mL^−1^), while the extracellular PC increased progressively to a maximum level of 3.91 mg mL^−1^ at SA 1 mM (Figure 3b), nearly 2-fold more than mock 1. In fact, SA extremely and surprisingly affected the exudation of nearly the whole amounts of crocin and phenolics biosynthesized inside saffron cells into the spent media. No significant differences were observed between crocin and PC amounts obtained from mock 0.1 and untreated control cells (data not shown).

### 2.3. SA Effect on Subcellular Compartmentalization

**Figure 4:**
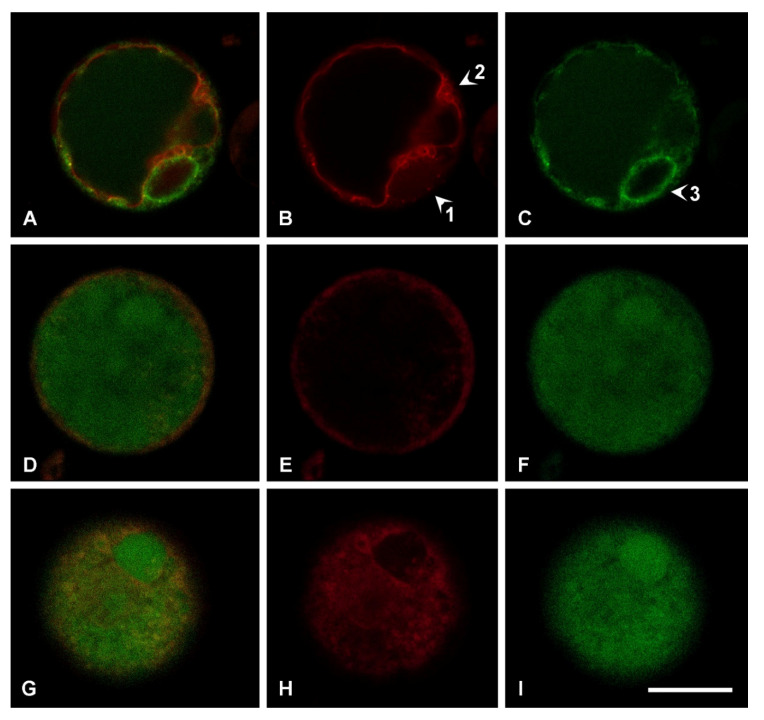
Protoplasts transiently transformed with vacuolar markers. (**A**) Protoplast expressing RFP-SYP51 and GFPgl133Chi; (**B**) the red RFP-SYP51 signal allows to appreciate tonoplast but also Trans Golgi Network (TGN) (arrow 1) and small vacuoles in the proximity of the central one (arrow 2); (**C**) the green GFPgl133Chi signal is distributed between the Endoplasmic Reticulum (ER) and the central vacuole, the nuclear ring is also well visible (arrow 3). (**D**) Equatorial optical section of a protoplast expressing AleuRFP and GFPgl133Chi; (**E**) the red AleuRFP signal is not efficiently labeling the central vacuole; (**F**) the green GFPgl133Chi signal is completely in the central vacuole. (**G**–**I**) Polar optical section of the same protoplast shown in D–F and confirming the same distribution of fluorophores. Scale bar 20 μm.

The cellular suspension can be reduced to protoplasts by digesting the wall using the same enzymatic mixture classically used for tobacco or Arabidopsis leaf tissues [23]. The resulting protoplasts are autofluorescent due to the high content of complex metabolites but can also be transiently transformed with fluorescent reporter proteins to characterize their compartmentalization. The vacuolar marker GFPgl133Chi was transiently expressed and evidenced its usual pattern [24]. Within 20 h GFP fluorescence was visible in the central vacuole (Figure 4) as well as in the Endoplasmic Reticulum (ER), revealed by the observation of the nuclear envelope (Figure 4A,C, evidenced by arrow 3). With time the ER labeling decreased. This marker was co-expressed with the Trans Golgi Network (TGN) and tonoplast marker RFP-SYP51 [25]. It showed that TGN is well visible (Figure 4B, arrow 1) and, in addition to the central vacuole, several small compartments in close contact but separated from the tonoplast are also visible (Figure 4B, arrow 2). GFPgl133Chi was also co-expressed with the lytic vacuole marker AleuRFP [26]. When GFPgl133Chi is completely exported from the ER and labels the central vacuole, AleuRFP still has a granulous distribution, evidencing that it′s sorting to the vacuole is not yet complete (Figure 4D–G).

**Figure 5:**
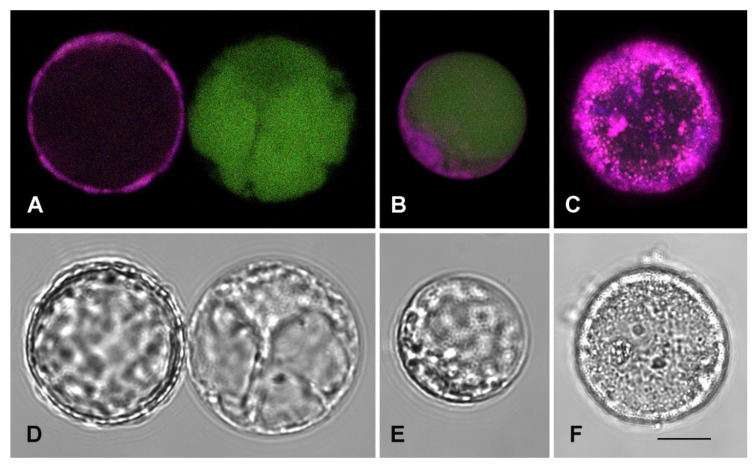
Autofluorescent protoplasts from saffron calli. (**A**) Two protoplasts showing opposite fluorescent patterns, on the left one with only orange-to-red emission (magenta color) in the cytosolic organelles (and little fluorescence in the central vacuole), the other on the right with only green emission in the central vacuole. (**B**) Protoplast from the same population with both emission patterns. (**C**) The most common fluorescent pattern showed by protoplasts treated with SA 1 mM. (**D**,**E**) Cells in control conditions do not show signs of stress. (**F**) SA treatment causes visible stress in a protoplast. Scale bar 10 μm.

We observed that protoplasts had compartmentalization similar to parenchimatic cells with a unique central vacuole, but the population was anyhow heterogeneous in relation to autofluorescence. The autofluorescence generated by the excitation of 488 nm, emitted in the entire spectrum with relevant fluctuations. To visualize these fluctuations (not quantified), we show in Figure 5 that different emission patterns can be observed in control conditions (Figure 5A,B). Differences were probably originated from the different age of cells. Since cell suspension was elicited with SA to stimulate crocin production, 1 mM SA treatment was also applied to protoplasts. The treatment caused tremendous stress to protoplasts (Figure 5C,F) and induced death within a few hours. This observation suggested that SA treatment was determining the accumulation of metabolites in the first hours after treatment since cells were not increasing in number and were probably dying.

### 2.4. Optimization of the Elicitation Time and Harvest Time for Metabolite Productions

**Figure 6:**
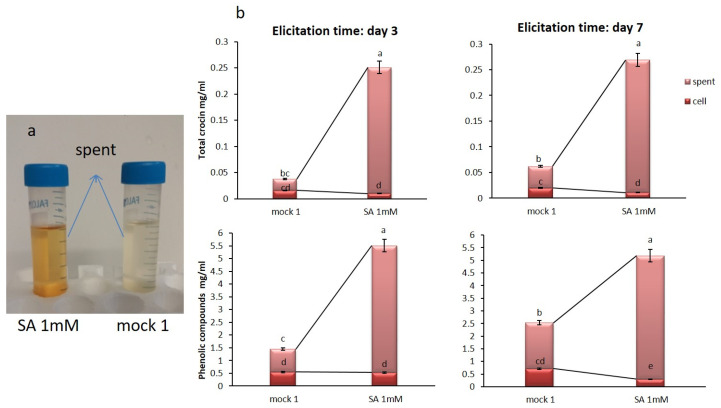
The exudation of saffron metabolites into the spent medium from cells treated with SA 1 mM after 24 h post-elicitation (**a**); total crocin and PC analysis after 24 h post elicitation at two different elicitation times, day 3 and day 7, in cells treated with SA 1 mM and mock 1, measured intra and extracellularly (**b**). Values without a common letter are statistically different (*p* < 0.05).

Once we had observed the SA (1 mM) lethal effects on saffron protoplast within 24 h, we also evaluated total phenolics and crocin production 24 h after treatment. At first, the SA application was done conventionally at the time of the beginning of the exponential phase of the growth cycle of Saffron cells on day 3. The results evidenced that nearly the entire metabolite exudation into the spent medium was obtained within 24 h (Figure 6a). The amount of nearly 0.25 mg mL^−1^ total crocin and 5 mg mL^−1^ phenolics were obtained after SA 1 mM elicitation at day 3 and 24 h post elicitation time in the spent medium of saffron cells. Then, to obtain as much biomass as could be achieved, we also applied SA elicitor to saffron cell suspensions at the end of the exponential phase on day 7. No significant differences were observed between the metabolite yields (amount/biomass) from cells treated on day 7 compared to those obtained from cells treated on day 3 (Figure 6b). The advantage of having larger biomass was then evident.

### 2.5. Antioxidant Activity of Saffron Cell Culture Extracts

**Figure 7:**
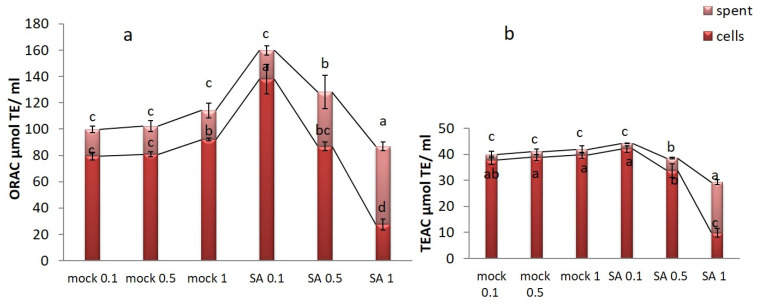
Antioxidant activity of methanol: water extracts of saffron in mock and SA (mM) treated cells intra (cells) and extracellularly (spent medium) measured by; (**a**) Oxygen radical absorbance capacity (ORAC); (**b**) Trolox equivalent antioxidant capacity (TEAC) assays. Common letter in each group indicates no significant differences (*p* < 0.05).

To measure antioxidant activity in cell extract and spent medium of saffron cell cultures, in mock and SA-treated, three different assays were applied: oxygen radical absorbance capacity (ORAC), trolox equivalent antioxidant capacity (TEAC), and 1,1-diphenyl-2-picrylhydrazyl (DPPH). ORAC assay measures antioxidant activity by hydrogen atom transfer and provides a comprehensive analysis of a test sample’s antioxidant activity in biological fluids, cells, tissues, and natural extracts [27]; TEAC assay is based on a single electron transfer activity [28]. These two assays could be normalized to Trolox equivalent units to quantify the composite antioxidant activity of the samples. Further, DPPH activities in cell extracts and their spent media were represented as % Inhibition (calculated by the electron-donation ability of the samples) and IC 50 value (the sample concentration required to inhibit DPPH free radical by 50%) [29]. In Figure 7, the antioxidant activity is reported as μmol of Trolox equivalents (TE) per mL (µmol TE mL^−1^) of saffron. The treatment with 0.1 mM SA showed the highest antioxidant activity in the spent medium of saffron cell cultures being 44. 22 and 159.88 µmol TE mL^−1^ for TEAC and ORAC assay, respectively. Moreover, ORAC and TEAC assays showed enhanced values of antioxidant activity in the spent medium of SA-treated saffron cells which correlated with increased amounts of metabolites like crocin and phenolics. In the case of DPPH assay, 20, 30, 40, and 50 µg mL^−1^ of saffron cell extracts and their spent media were evaluated as the optimum concentration for antioxidant activity, bleaching the purple color to yellow color. Inhibition percentage (I %) and IC50 are reported in Table 1. In the present study, the three applied assays showed a similar trend. In the case of DPPH assay, the lowest value of 19.47 IC 50 radical scavenging activity was obtained from cell extracts treated with 0.1 SA and their spent media indicating the most effective antioxidant activity, compared to the positive control ascorbic acid having IC50 6.79.

### 2.6. Effects of Saffron Cell Suspension Extract on Breast Cancer Cells

**Figure 8:**
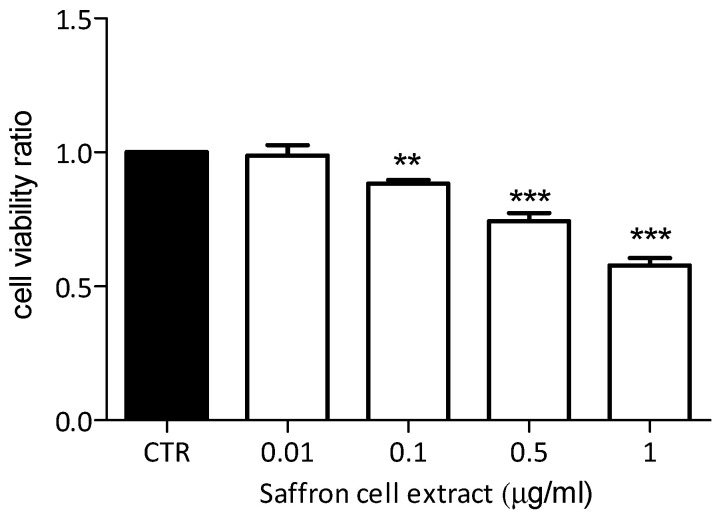
Cell viability of human breast cancer cell line MDA-MB-231 after 72 h treatment with saffron aqueous extracts at different concentrations. Data represent mean values of independent experiments performed in six technical replicates, ± standard deviation. CTR, control. ANOVA statistic test followed by Dunnett’s post-test was used to compare each treatment with the control (** *p* < 0.01 and *** *p* < 0.001).

Water-soluble extract of saffron cells was examined for its effect on the viability of a well-characterized human breast cancer cell line MDA-MB-231. As shown in Figure 8, almost all used concentrations of saffron cell extract (1, 0.5, and 0.1 µg mL^−1^) showed significant inhibitory effects on cell viability after 72 h from their application, compared to untreated cells (control, CTR). Furthermore, this activity seems to be directly proportional to the concentration used, showing a stronger inhibitory effect at higher concentrations (1 and 0.5 µg mL^−1^, *p* < 0.001) and a slight but significant activity at 0.1 µg mL^−1^ (*p* < 0.01).

## 3. Discussion

A saffron cell suspension culture was established from calli optimized for their production of crocin and phenolic compounds. Intending to set an appropriate strategy to produce extracts with antioxidant and cell inhibitory activity, we here characterized the effect of cell elicitation by SA. Saffron is a sterile triploid plant of the Iridaceae family which is propagated only through cormlet production as the multiplication rate is quite slow, so concerns have risen due to the decreased production of saffron during the last two decades [30]. Many researchers studied the propagation of saffron through in vitro cultures such as shoot regeneration [31,32] and cormlet production [33,34], or the production of saffron typical metabolites through the induction of callus cultures [35] and stigma like structures [36] from various saffron explants; however, there are no valid reports on saffron cell suspension culture establishment and characterization so far. Since saffron metabolites mostly crocin are very precious, therefore the availability of plant suspension cultures, for the production of saffron valuable bioactive compounds is an important improvement in satisfying the great demand for this valuable product for the food industry. The availability of plant cell cultures for the production of specific metabolites is beneficial for providing a continuous supply of phytochemicals in controlled and axenic conditions, as a valuable alternative to the extraction from plants grown in the field under environmental and seasonal strain [9].

In this study, a saffron suspension cell culture was established and characterized as for the in vitro production of crocin and phenolics. In our system, SA was confirmed to be a valuable elicitor not only in terms of significant metabolite induction but also for the induction of metabolite exudation into the spent medium. At any applied concentration, SA significantly increased the extracellular amounts of apocarotenoid crocin and phenolic compounds which decreased in cell extracts. At the same time, it was evident that the reduced intracellular content of these metabolites could also be explained by the dramatic changes in cell viability due to the SA application. The growth rate of saffron cell suspension was significantly affected, decreasing along with the increase of SA concentration within a 10-day growth period, compared to the mock cells. In this regard, our results are in agreement with those collected with other cell cultures such as *Taxus baccata* [37], *Salvia miltiorrhiza* [38], *Hypericum perforatum* [39], and *Polygonum minus* [40], where cell growth was also inhibited after elicitation by SA. Indeed, SA elicitor application at the highest concentration dramatically stressed the saffron cell culture, causing cell death within 24 h. In this time, the elicitor also increased the production of saffron natural metabolites especially crocin, and caused their exudation into the culture medium. This behavior makes SA a promising elicitor to be used in a possible scaling-up procedure for bioreactor production of saffron bioactive compounds [41,42].

In plant cell cultures, phenylpropanoid compounds are those metabolites that are mostly affected by elicitors such as SA, commonly associated to a rapid and temporarily increased activity of the key biosynthetic enzymes phenylalanine ammonia-lyase (PAL) and chalcone isomerase (CHI) [43,44,45]. Enhanced levels of PC after SA treatment were reported in suspension cultures of *Vitis vinifera* and *Polygonum multiflorum* [20,46], as well as in *Thevetia peruviana* [19]. It seems that exogenous SA uptake into the cell induces resistance responses through the activation of anti-oxidative enzymes and eventually produces ROS species like H_2_O_2_. These play an important role as a second messenger in SA signaling pathways leading to the production of secondary metabolites in plant cell cultures [38,47]. In the established saffron suspension cell culture, we evaluated that 1 mM SA was the most effective treatment inducing crocin and phenolic biosynthesis. Besides, it stimulated the exudation of the bioactive compounds. In protoplasts from the same suspension culture, we observed that the elicitor SA induced strong cellular stress leading to cellular death within 24 h post elicitation time.

The protoplasts derived from saffron cells provided interesting information about the compartmentalization of cells in suspension culture, being representative of the original cells [24]. We transiently transformed the protoplasts with the vacuolar marker GFPgl133Chi. This marker consists of a GFP carrying the vacuolar sorting determinant of tobacco ChitinaseA and characterizes vacuoles with the less acidic lumen and less pronounced lytic activity [46]. Its sorting pathway was recently shown to bypass the Golgi apparatus [24]. It showed that protoplasts from cells always had a unique well-labeled central vacuole. GFPgl133Chi was co-expressed with the TGN and tonoplast marker RFP-SYP51, based on the fusion of RFP with the Arabidopsis SNARE SYP51 [25,48]. It revealed that in addition to TGNs and the central vacuole tonoplast, several small compartments could be observed around the central vacuole. GFPgl133Chi labeling did not appear evident in these small compartments and, since it follows a Golgi-independent pathway alternative to SYP51 sorting, we consider this observation as a confirmation that these are separated from the central vacuole. Then we co-expressed GFPgl133Chi with a second soluble vacuolar marker, AleuRFP [25,26] that follows the same sorting pathway of SYP51 [25]. This marker is considered to label lytic vacuoles following the classic sorting pathway dependent on the Golgi apparatus. The marker is the product of the fusion between the N-terminal vacuolar sorting determinant of the protease Aleurain from barley and RFP [49]. Aleurain is recognized by the well-known vacuolar sorting receptors VRS1 and its sorting is usually more efficient than GFPgl133Chi’s sorting [24] and all previous literature comparing the two markers). We also observed that protoplasts from cells are rich of autofluorescence and that SA triggers cell death through the formation of a multitude of small autofluorescent compartments, often observed during stress and cell death. These observations suggested that protoplasts from cells had a vacuolar system with a central vacuole characterized by a marker of neutral vacuoles [24,50] and several small peripheric compartments characterized instead by the marker usually associated with proteases sorted through the Golgi apparatus. These peripheric compartments may be associated with those elicited by stress and SA.

We then demonstrated, by metabolite quantification after 24 h SA application, that also the metabolite production was induced immediately after elicitation. In agreement with our study, Mendoza et al. [19] also evidenced that SA affected cell suspension cultures of *Thevetia peruviana* obtaining an increased value by 21.41% of phenolic compounds during the first hours after elicitation. No significant differences were observed between the metabolite yields, in terms of amount/biomass, from cells treated on day 7 compared to those obtained from cells treated on day 3, thus the application of SA to saffron cells on day 3 of the growth cycle could be considered more efficient for biotechnological purposes in terms of time saved but the attainment of the maximum biomass after 7 days of cell growth is preferable to optimize the bioproduction effort.

SA 1 mM led to the highest production of saffron bioactive compounds, likely as stress and defense response, and eventually their exudation into the spent media, while SA 0.1 mM showed the highest effects on antioxidant activity inside the cells. The latter can be evaluated as the optimal concentration in terms of induction of intracellular bioactive antioxidants in saffron cells. Investigation of saffron antioxidant and free radical scavenging activities has been conducted by many researchers but differing results were obtained due to the different origins, methods, and solvents used for extraction [51,52]. In Indian *Crocus sativus* L. the free radical scavenging activities were tested at concentrations ranging from 100 to 500 µg mL^−1^ with the highest activity in stigma extract of 70% followed by 79.6% in callus extracts [53]. Karimi et al. [54] reported a maximum of 210.79 µg mL^−1^ IC 50 of methanolic stigma extracts as compared with 255.44, and 299.44 µg mL^−1^ for boiling water and ethanol extract, respectively. They identified 6.5 mg gallic acid equivalent g^−1^ DW Phenolic Content and 5.8 ± 0.12 mg rutin equivalent g^−1^ DW flavonoid content in stigma extract as well. Further, the IC50 value of ethanolic extract of stigma was reported at 207.16 µg mL^−1^ by Baba et al. [55], while aqueous extract of Italian saffron showed the low IC50 value of 3.76 mg DW [56]. Since the most abundant metabolites of saffron are crocin and safranal, DPPH radical scavenging activity has been proved to be attributed to these two main bioactive compounds [57,58]. In the present study, saffron cells evidenced a considerable antioxidant activity under SA treatment which could be related to the high production of phenolic content and crocin. Phenolic compounds of plants have been identified to directly contribute to their antioxidant value [59], as well as the correlation between them and antioxidant activity have also been corroborated [60,61,62].

Saffron crocin and safranal have been studied for their ability to reduce cellular proliferation in different models including lung (A549), colon (HCT-116), breast (T47d), and prostate (PC-3) cancer cell lines [63]. For this reason, to test whether the saffron extract obtained from our cell suspension can reproduce this biological action in vitro, we determined the effects on a breast cancer human cell line. We showed that the aqueous extract of saffron cell suspensions showed a significant inhibitory effect on the growth of the aggressive breast cancer MDA-MB-231 cells [64], in a dose-dependent manner, even at the concentration of 0.1 µg mL^−1^. This is an issue of great importance since we clearly described the approach used to obtain a fresh extract to be used on cells.

## 4. Materials and Methods

### 4.1. Establishment of Cell Suspension Cultures and SA Treatment

Cell suspension cultures were established by adding 2 g of friable calli to 100 mL Erlenmeyer flasks containing 3–5 mL of liquid MS medium with the addition of NAA 3 mg L^−1^ and TDZ 1 mg L^−1^), incubating on a rotary shaker (120 rpm) in the dark at 25 °C. The final volume of 25 mL was reached gradually in one month. Once the suspension culture was established, subcultivation was performed every 10 days. For subcultivation, 5 mL of grown suspension was transferred into 50 mL of fresh MS medium. The biomass accumulation was determined by measuring the fresh weight of the cultures at every 2 days. Each point of the growth curve represents the mean of three independent replications (flasks). Staining of the cells with fluorescein diacetate was done for controlling the cell viability [65].

SA at three different concentrations of 0.1, 0.5, and 1 mM was applied to examine the elicitation effect on biomass accumulation and metabolite production. SA was first dissolved in methanol, diluted with deionized water, and filter-sterilized before adding to the culture medium. The same amount of filter-sterilized methanol as used for each concentration of SA was added to the cell suspension cultures representing mock 0.1, 0.5, and 1. Ten-day old cells were filtered and 2 g fresh weight (FW) of them was transferred to 50-mL flasks containing 30 mL MS medium with 3 mg L^−1^ NAA + 1 mg L^−1^ TDZ. Flasks were incubated on a rotary shaker at 120 rpm and maintained at 23–25 °C in the dark. Cells were elicited with SA and mock solutions, unless differently specified, after 3 days of culture and maintained in the presence of treatment for further 4 days. Then, cells were centrifuged at 4000× *g* for 10 min (Beckman Allegra 22R, Beckman Coulter, Brea CA, USA), lyophilized (Labconco, Kansas City, MO, USA), and kept at −20 for metabolite analysis. The supernatant was used as the spent medium for further analyses.

### 4.2. Extraction of Crocins from Cells

The extraction of crocins was accomplished according to Hoshyar and Bathaie [66]. The lyophilized cells (1 g FW) were extracted with 5 mL of methanol-water (50:50% *v*/*v*) for 24 h under magnetic stirring in dark at 4 °C and centrifuged at 4000× *g*. The supernatant was collected and filtered through a nylon membrane (Acrodisc 13, 0.45 µm pore size, and 13 mm diameter), and injected as the cell extracts into the HPLC device (Agilent-1290 infinity Agilent Technologies, Waldbronn, Germany).

### 4.3. Extraction of Crocins and Phenolic Compounds from the Spent Medium of Suspension Cultures

The extraction of crocetin esters (crocins) from the spent medium was performed using the method of Schweiggert et al. [67], used for the extraction of free carotenoid and carotenoid esters with some modifications. Briefly, the 30 mL of discarded medium was washed with a mixture of 50 mL acetone: hexane (1:1, *v*/*v*) containing ascorbic acid as an antioxidant by a glass separatory funnel. After shaking, 50 mL of sodium chloride solution (10%, *w*/*v*) was used to wash the solution, followed by twice with 50 mL of water to remove acetone. The aqueous phase was re-extracted with ethyl acetate until it was colorless then evaporated with a vacuum pump. The residue was dissolved in a 5 mL mixture of methanol-water (1:1, *v*/*v*), membrane-filtered (0.2 mm), and used for crocin quantification by HPLC analyses as well as total phenolic content spectrophotometrically (Infinite M200 Tecan, Männedorf, Switzerland).

### 4.4. Extraction of Phenolic Compounds from Cells

The extraction of phenolic compounds from saffron cells was done according to Ranganna [68]. The cells were centrifuged at 4000× *g* for 10 min and then filtrated through nylon filters (30-µm pore size). Fresh cells (0.5 g) were homogenized with methanol 80% and kept for 3 h in a water bath at 70 °C. The extract was then centrifuged at 9000× *g* for 15 min at room temperature. The methanolic extract was discarded and brought up to volume for the analysis.

### 4.5. Total Phenolic Content

The total phenolic content of the samples was quantified according to the Folin-Ciocalteu method [69]. For quantification of intra and extracellular phenols, 1 mL of each sample extracted from cells and spent media were mixed with 2.5 mL Folin-Ciocalteu reagent (diluted with distilled water 1:10 *v*/*v*), 2 mL of 7.5% sodium carbonate and left in the dark at room temperature for 2 h. The absorbance of samples was measured at 725 nm against a blank. Total phenolic content was expressed as gallic acid equivalent (mg g^−1^ fresh weight) based on a gallic acid standard curve.

### 4.6. HPLC-DAD Analysis

Crocin was quantified by an auto-sampler HPLC device (Agilent-1290 infinity Agilent Technologies, Waldbronn, Germany) equipped with a C18 column (250 mm × 4.6 mm) and a multiple ultraviolet (UV) wavelength photodiode array detector. The elution was performed with a linear gradient of methanol: water: acetonitrile (50:42.5:7.5 *v*/*v*) as a mobile phase, a flow-rate of 1.0 mL min ^-1^ and a maximum elution time of 30 min at room temperature and 440 nm. We used 2 crocin standards one including all identified crocetin esters (Sigma-Aldrich, Taufkirchen, Germany) for identification, and the other for pure α-crocin for the quantification of the crocetin esters (PhytoLab, Darmstadt, Germany).

### 4.7. Protoplasts Preparation, Transformation, and Analysis

*C. sativus* protoplasts were prepared as previously described for Nicotiana tabacum leaves and other plant material such as cell suspension cultures [23,70] from cells harvested from 3 or 7 day-old suspension culture. The observations evidenced no differences among protoplasts at the end of the process. Since the experimental procedure was strictly dependent on a good amount of cells, more experimental replicas were performed with cells grown 7 days available with higher biomass. The three vacuolar markers were previously characterized and used in several studies: GFPgl133Chi [24], RFP-SYP51 [25,49], AleuRFP [26,50]. Protoplasts were examined with a confocal laser microscope (LSM 710 Zeiss, ZEN software, GmbH, Jena, Germany). GFP was detected within the short 505–530 nm wavelength range, assigning the green color, RFP within 560–615 nm assigning the red color. Excitation wavelengths of 488 and 543 nm were used. Green autofluorescence was detected within the 505–560 nm wavelength range, red autofluorescence was detected above 650 nm. The laser power was set to a minimum and appropriate controls were made to ensure there was no bleed-through from one channel to the other. Images were processed using Adobe Photoshop 7.0 software (Mountain View, CA, USA).

### 4.8. Trolox Equivalent Antioxidant Capacity (TEAC) Assay

TEAC assay was done according to Re et al. [28]. TEAC assay is based on the scavenging of radical cation ABTS^•+^ (2,2′-azino-bis (3-ethylbenzthiazoline-6-sulfonic acid) by antioxidants, ABTS^•+^ is green in color and can be measured by absorbance at 734 nm. For the preparation of ABTS^•+^ radical cation stock solution, we used the concentration of 7 mM ABTS and 2.45 mM potassium persulfate, which was mixed and kept in dark at room temperature for 12–16 h before use. The fresh ABTS radical cation working solution was obtained with a dilution of 7 mM ABTS^•+^ stock solution with PBS (pH 7.4) to an absorbance of 0.70 (±0.02) at 734 nm on the plate reader equilibrated at 30 °C. The assay was adjusted to a 96-well format (Costar, 96-well clear round bottom plate, Corning). 210 µL final volume (10 µL of the sample or Trolox standard solutions (from 0 to 25 μM) with 200 µL diluted ABTS^•+^ solution) was prepared to each well of the microplate, reading the absorbance at 734 nm after 10 min using a microplate reader (Infinite M200 Tecan, Männedorf, Switzerland). For the Trolox reference standard, the percentage inhibition of absorbance at 734 nm was plotted and used along with the solvent blanks in every assay.

### 4.9. Oxygen Radical Absorbance Capacity (ORAC) Assay

ORAC assay was performed according to Davalos et al. [27] method using a 96-well format (Costar, 96-well clear round bottom plate, Corning). Assay final volume of 200µL was carried out in 75 mM phosphate buffer (pH 7.4). The solutions of the antioxidant (20 µL) and fluorescein (120 μL; 70 nm, final concentration) were prepared in the well of the microplate and preincubated for 15 min at 37 °C 60 µL (12 mM, final concentration) of 2,2′-Azobis-(2-methylpropionamidine) dihydrochloride (AAPH) solution was added and placed immediately in a microplate reader (Infinite M200 Tecan, Männedorf, Switzerland), and the fluorescein was recorded (excitation and emission wavelengths of 485 and 527 nm, respectively) every minute for 1 h. Three dilutions of samples were used in each assay and for every sample, three replications were used. We used a blank of fluorescein and AAPH in each assay. Diminution curves of fluorescence (intensity vs. time) were registered, followed by the calculation of area between the two Diminution curves (with or without antioxidant). Subtracting the blank amount from that of the sample or standard was acquired for the pure area under the curve. A standard curve of Trolox solution using different concentrations (1–6 μM) was used in every assay. Final ORAC values were represented as μmol Trolox equivalents (TE) mL^−1^ of extract.

### 4.10. 1-Diphenyl-2-picrylhydrazyl (DPPH) Radical Scavenging Assay

DPPH assay was performed according to Blois [29] method. The antioxidant ability of saffron cell extracts treated with SA and their spent media were measured by their scavenging activity in terms of hydrogen atoms or electron-donation ability to the stable free radicals of DPPH solution. Three mL DPPH methanolic solution was added to 1 mL samples containing different concentrations of saffron cell extracts and their spent media (20, 30, 40, and 50 µg mL^−1^) and kept in the dark at room temperature. After 30 min, the absorbance was read at 517 nm against the reagent blank. Ascorbic acid was used as the positive control in the same concentrations as the samples.

The free radical scavenging activities of the samples were determined as inhibition (%) and were calculated according to the following formula:

Percent % inhibition of DPPH activity = [(A0 − A1)/A0)] × 100%(1)

A0 = DPPH solution absorption, A1 = DPPH solution absorption after sample addition.

IC 50 value was calculated by plotting % inhibition versus concentration of samples curve to determine the optimal concentration of the antioxidants for decreasing the initial DPPH concentration by 50%, using the GraphPad Prism software.

### 4.11. MTT Assay 

The concentrations of 1, 0.5, 0.1, and 0.01 µg mL^−1^ saffron extracts were prepared from 0.5 g lyophilized saffron cell suspensions which were grown for 10 days beforehand. The extraction was conducted as described above, but extracts were dissolved in only distilled water at 25 °C and kept at 4 °C until the analysis. Saffron aqueous extract effects were tested against the breast cell line MDA-MB-231. Cells were cultured in DMEM medium (4500 mg L^−1^ glucose) supplemented with 10% Fetal Bovine Serum (FBS), 100 U/mL penicillin, and 100 μg mL^−1^ streptomycin at 37 °C in an atmosphere of 5% CO_2_. For determining cell viability, the MTT (3-[4,5-Dimethylthiazol-2-yl]-2,5-diphenyltetrazolium bromide) assay was performed as previously described [71]. Cells were plated at 5 × 10^3^ cells per well in a 96-well plate and allowed to adhere to the plate overnight under the growth conditions described above. After 72 h of treatment, the culture medium was aspirated and the MTT solution was added, according to the manufacturer’s instructions (SIGMA). After one hour of incubation, the MTT solution was removed and 100 µL of DMSO was added to dissolve MTT–formazan crystals. The absorbance of the converted dye was measured at a wavelength of 570 nm using an iMark microplate reader (BIORAD, Hercules, CA, USA). Data were normalized to the control value and expressed as mean ± standard deviation.

### 4.12. Data Acquisition and Statistical Analysis

All experiments were repeated 3 times and analyzed by one-way analysis of variance (ANOVA) using the SPSS-22 software. Differences among the means were tested at the significance level of *p* < 0.05 with the Duncan test. Statistical analyses on MTT data was performed by GraphPad software (Prism 5.03). Analysis of variance (ANOVA) and Dennett’s post-hoc-test was applied to compare control with all treatments, Differences were considered statistically significant for values *p* < 0.05. Data are represented as mean ± standard deviation (SD).

## 5. Conclusions

We obtained from style-derived calli a cell suspension culture of saffron (*Crocus sativus* L.) producing relevant quantities of crocin together with total phenolics. Interestingly, saffron cell extracts also showed significant inhibitory effect on the viability of a breast cancer cell line. Both crocin and phenolics accumulation patterns after SA elicitation indicated their contribution to the increase of antioxidant activity. We investigated at a subcellular level the status of suspension cells, using protoplast transient transformation with heterologous vacuolar markers followed by confocal microscopy. We learned that vacuolar morphology was similar in all the cells, but endogenous autofluorescence revealed heterogeneity when considering metabolite production. Since a remarkable increase of total crocin and phenolic compounds were quantified in the spent medium of cell suspensions under SA elicitation, we performed microscopic observation of the effect induced by the highest SA concentration (1 mM). We observed that it caused stress and death within 24 h after elicitation when the exudation of high amounts of bioactive compounds occurred. Most of the crocin and phenolics production occurred during the SA elicited cell apoptosis within the first 24 h so that the biotechnological exploitation of SA elicitation may take place at any moment, but at the end of the growth curve, at day 7, the optimal biomass accumulation is obtained. On the other hand, reducing the elicitation time to 24 h before harvesting could be considered a high-valued innovation in terms of saving time in scaling up the process for metabolite production independently from saffron cell growth rate.

## Figures and Tables

**Table 1:** Inhibition percentage (I %) and IC50 (µg mL^−1^) of free radical scavenging activities from DPPH assay in saffron mock and salicylic acid (SA) treated cells at different concentrations of both cell extracts and their spent media.

Sample	I%				IC 50 (µg mL^−1^)
	20 µg mL^−1^	30 µg mL^−1^	40 µg mL^−1^	50 µg mL^−1^	
Ascorbic acid	66	81.66	90.33	97.66	6.79
0.1 mock	26.13	33.16	41.62	49.83	55.41
0.5 mock	27.72	34.92	41.59	48.91	54.19
1 mock	29.72	37.49	43.10	51.51	49.37
0.1 SA	39.19	56.06	73.23	85.13	19.47
0.5 SA	26.53	39.58	58.51	81.53	31.25
1 SA	19.31	30.68	50.65	72.79	42.77
